# The Effect of Cultivation Method of Strawberry (*Fragaria x ananassa* Duch.) cv. Honeoye on Structure and Degradation Dynamics of Pectin during Cold Storage

**DOI:** 10.3390/molecules25184325

**Published:** 2020-09-21

**Authors:** Magdalena Drobek, Magdalena Frąc, Artur Zdunek, Justyna Cybulska

**Affiliations:** Institute of Agrophysics, Polish Academy of Sciences, Doświadczalna 4, 20–290 Lublin, Poland; m.drobek@ipan.lublin.pl (M.D.); m.frac@ipan.lublin.pl (M.F.); a.zdunek@ipan.lublin.pl (A.Z.)

**Keywords:** pectin, structure, cold storage, quality, strawberry, cultivation

## Abstract

The high quality and long shelf life of strawberry fruit are largely dependent on the cultivation method. The goal of this experiment was to study the effect of different cultivation methods on molecular structure and rheological properties of pectin extracted from strawberry quality parameters during cold storage. Three methods of cultivation of strawberry cv. Honeoye were tested: organic cultivation on raised beds, organic cultivation with the flat-planted method and conventional cultivation with the flat-planted method. The nanostructure of pectin (AFM), its chemical structure (FT-IR) and rheological properties were studied. The fruits were also tested by size, dry matter, firmness, acidity and the content of soluble solids, anthocyanin, phenolics, vitamin C and galacturonic acid. Pectin isolated from organic strawberries was more rapidly degraded than conventional strawberry pectin, which limits the possibilities for their processing and use as gelling or stabilizing agents at 20 °C. The differences in fruit quality were particularly noticeable with respect to the anthocyanin content, which was significantly higher for organic strawberry. The organic fruit also had better sensory properties because of its lower acidity and higher soluble solid content (SSC). These and other results from this experiment showed that strawberries produced by organic farming methods had better biochemical properties compared to conventional fruit; however, pectin transformation undergone faster limits their further technological applications.

## 1. Introduction

Strawberries are a popular soft fruit around the world because of their taste and nutritional value. The fruit yield in the European Union in 2011 increased by an average of 20% compared to 2010 (9766.3 kg ha^−1^) and remained stable until 2018 (12,189.9 kg ha^−1^) [1]. The increase in yield is a consequence of the increase in knowledge about cultivation, the availability of new varieties and the development of agricultural technologies. However, farmers still have to deal with agrometeorological conditions. Spring chill is a factor in slowing vegetation growth. Delayed flowering, low air temperature and rain delay the pollination of flowers by bees. Excess humidity provides a good environment for the development of bacteria, viruses, protozoa and worms, while water shortage results in the rapid inhibition of the growth of aerial parts and interferes with the photosynthesis and transpiration processes. All of these processes can cause a reduction in fruit quality [2].

Food quality includes many attributes such as sensory, mechanical and functional properties, chemical constituents and nutritive values [3]. In the case of strawberries, their appearance (colour and biometrical characteristics), firmness, and chemical composition have the most influence over consumers’ preference [4]. First of all, the colour and hardness of the fruit are important. Firm, hard fruit is easier to store, and glossy skin attracts the attention of consumers. Firmer fruit ismore resistant to mechanical damage, which is inevitable during harvesting, transport and storage. Harder fruit is also more desirable to consumers [5], but also less susceptible to fruit rot caused, for example, by *Botrytis cinerea* [6]. Next, taste is important, which is affected mainly by acidity and soluble solid content and to some extent by the content of vitamins, macro- and microelements, anthocyanin and phenolic acids, which give the fruit health benefits [7]. Due to the content of bioactive compounds, strawberry fruit has antioxidant, anticancer, anti-inflammatory and anti-neurodegenerative effects [8,9].

The sensory texture and firmness of the fruit largely depend on the mechanical strength and stiffness of the cell walls [10,11,12]. Cell walls, made of glycoproteins and polysaccharides such as cellulose, hemicellulose and pectin, provide protection against external factors, which has a direct impact on the quality of the fruit. The pectin content of cell walls is up to 50% [10,11,12]. During fruit development and ripening, cell wall polysaccharides, and especially pectin, undergo the most pronounced changes associated with the action of pectinlytic enzymes [10]. It is assumed that these enzymes change the structure of the cell wall, leading to a loss of fruit quality and a shortening of their shelf life [13,14]. The industrial use of strawberries often depends on the pectin content and its ability to gel, which results from rheological properties [15,16,17]. Rheological studies enable the determination of organization and behaviour of pectin-containing fluids [13,18]. Thus, the structure of pectin on a molecular level is an important factor that considerably influences the quality of fruits and its industrial use [15,19].

Ensuring the quality of strawberries starts in the field with the choice of variety and method of cultivation. There are two main production systems for strawberries: conventional and organic. Conventional crops require less investment than organic cultivation. However, it is known that the conventional system, characterized by the intense use of chemicals, generates residues, which may accumulate in agroecosystems and products [19]. In the case of crops under an organic production system, the greatest financial burden is associated with the labour costs, biofertilizers and special biological agents against pests and other pathogens causing diseases [20]. It should be emphasized that although crops under organic production have a lower yield in general in comparison with conventional cultivation, organic farming produces healthier, pesticide-free fruit [19]. Strawberry plants can be planted directly into the ground using the flat-planting method or raised beds. Cultivation on beds involves making beds from soil about 15–40 cm high and 80–90 cm wide, which is covered by a non-woven fabric and provides a water supply line and nutrients [20]. The advantage of growing in beds is, among others, a reduction in water evaporation, fewer weeds and better use of nutrients [20]. On the other hand, the purchase of agrotextile and water supply lines generates additional costs. However, fruit from crops grown in raised beds often do not come into contact with the soil and therefore are less susceptible to soil contamination, and consequently, it is easier to maintain their quality [21].

The aim of the experiment was to determine which cultivation system provides fruit of the highest quality and how cultivation methods change the pectin structure and fruit quality parameters during cold storage. The structure and properties of pectin polysaccharides were determined using FT-IR spectroscopy, AFM microscopy and rheology.

## 2. Materials and Methods

### 2.1. Crop Conditions

The test material was the strawberries cv. Honeoye from the same geographical region (southeastern Poland), but the plants from which the fruits were harvested were grown in three different fields: on raised beds (organic) and in flat-planted plots (organic and conventional) (Table 1). The cultivation on raised beds was located within an average distance of 16 km from the organic-cultivation–flat-planting and an average of 46 km from conventional-cultivation–flat-planting. Organic-cultivation–flat-planting was on average 32 km from conventional-cultivation–flat-planting. The crops were in the same climatic zone: temperate with cold, cloudy, moderately severe winters with frequent precipitation and mild summers with frequent showers and thundershowers. The soils belonged to the podzolic and fallow soils. The annual (2019) rainfall was 500–600 mm. The average air temperature in the month of harvest (June) was 22, and the monthly (June) rainfall was 40–50 mm. For cultivation on raised beds (organic), the strawberry plants were planted in two rows on beds made from soil. The height of the bed was on average 15 cm, and the width at the base was about 80–90 cm. Each bed was covered with black agrotextile, which prevented soil drying and weed growth. Under the agrotextile in the middle of the bed, a drip line was positioned to supply water, nutrients and biostimulants. The distance between the strawberry plants in the rows was on average 15–30 cm, the distance between the rows in the bed was 30–40 cm and the spaces between the beds were around 60 cm and were lined with straw. In the fields where the crops were grown using the flat-planting method (organic and conventional), the strawberry plants were planted in two rows directly into the ground. The spacing between the plants in a row was on average 15–35 cm, and the distance between the adjacent rows was about 30–40 cm. The spacing between the double rows averaged 80–100 cm. Organic fertilizers, biostimulants and biopreparations against pests and pathogens were used in both types of organic production systems—using raised beds and the flat-planting method throughout the entire cultivation period until harvest—while in conventional crop production using the flat-planting method, chemicals and other organic and biological substances were used (Table 1). A minimum of 5 kg of fruit was collected from each field. The fruits were collected together with peduncles at the state of maturity confirmed by breeders. For the three crops tested, the fruit was harvested the same day. The fruits were carefully placed in boxes and maintained at an appropriate temperature before being transported to the cold store on harvest day.

### 2.2. Cold Storage

**Figure 1:**
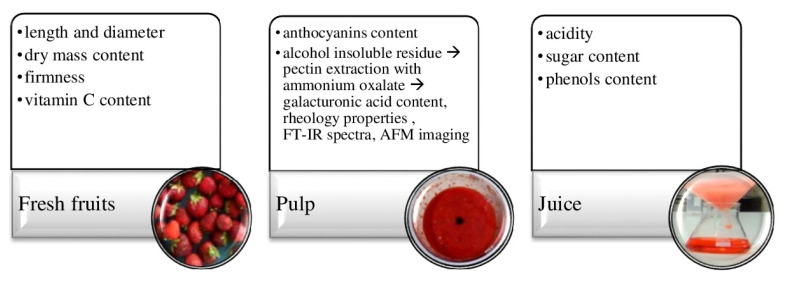
Scheme of quality testing of strawberries.

The fruits were placed in a cold store immediately after harvest (temperature 4–5 °C, humidity 70–80%). The cold storage reflected the varieties in which the fruit is stored by the producers before they are placed on the market. The experiment was carried out over a period of 12 d. The sampling days are indicated with the following abbreviations: T0—harvest day; T1, T2, T5, T6, T7, T9—consecutive days of cold storage; T12—last day of cold storage. The fruit sample (500 g of fruit taken randomly) was gathered on specific days, and the following tests were carried out: determination of fresh and dry matter, fruit dimensions, firmness, acidity, pH, soluble solid content (SSC), anthocyanin, phenols, vitamin C, galacturonic acid, rheological properties and a collection of FT-IR spectra (Figure 1).

### 2.3. Sample Preparation

In order to obtain juice, the fresh fruit was crushed and centrifuged for 10 min at 20,000× *g*. The pulp was obtained by blending fresh fruit using a homogenizer. The alcohol insoluble residue (AIR) was prepared according to the method reported by Renard (2005) [22] with modifications. Fresh fruit was homogenized and then rinsed with 96% ethanol and filtered using nylon filters. The residue was rinsed again with ethanol and filtrated until the sugar was discarded as determined by the Dubois test [23]. Finally, the residue was rinsed with 96% ethanol followed by acetone and dried at 40 °C.

#### Pectin Extraction with Ammonium Oxalate

Pectin extraction was carried out in accordance with the method proposed by Koubala et al. (2008) and Min et al. (2011) [24,25], with some modifications. Ten grams of AIR was mixed with 400 mL 0.25% ammonium oxalate. The mixture was incubated in a water bath (85 °C) for 1 h. After incubation, the suspension was centrifuged at 20,000× *g*, and the supernatant containing all of the pectin was collected. Three volumes of 96% ethyl alcohol were added to the supernatant and incubated for 24 h at 4 °C to precipitate the pectin. The mixture was centrifuged at 20,000× *g*, and the pellet was washed twice with 96% ethyl alcohol. The precipitate was then freeze-dried, and the pectin yield was counted.

### 2.4. Fruit Quality

#### 2.4.1. Fresh Weight

Fifty fruits were weighed and the results averaged.

#### 2.4.2. Length and Width

Immediately after harvesting, the fruit was weighed and measured with electronic callipers (Mega, Warsaw, Poland). The length (longer diameter) was measured from the base of the strawberry under the peduncle to the tip of the fruit, while the width (usually the shorter diameter) was the transverse measurement of the fruit at the thickest point. The measurements were carried out on fifty fruit from each crop, and the results were averaged.

#### 2.4.3. Dry Matter Content

The dry mass was determined according to standard PN ISO 1026:2000 [26]. For each crop, 0.5 g of strawberry was taken in triplicate and weighed on glass plates with an accuracy of 0.001 g. The material was dried in an oven at 105 °C for 24 h. The material was weighed again, and the result was noted; then it was placed back in the oven (105 °C, 2 h). After 2 h of incubation, the strawberries were removed and weighed again. If the weight was the same as it was after 24 h incubation, the dry weight was calculated.

#### 2.4.4. Firmness

Sample hardness was determined with a puncture test using a universal testing machine (Lloyd LRX, Lloyd Instruments, San Diego, CA, USA) equipped with a probe that had a width of 3 mm. The sample was penetrated to a depth of 6 mm at a speed of 20 mm min^−1^. The test was carried out on 25 strawberry fruits from one crop, and then the results were averaged. Firmness was defined as the maximum value of the force required to pierce the skin layer of the fruit [27].

#### 2.4.5. Acidity

Acidity was determined by the method proposed by Nunes et al. (1995) [28]. About 200 g of strawberries (in three replications) were taken from each crop, from which the juice was squeezed. The juice extraction method consisted of crushing the fruit, which was then centrifuged (20,000 × g, 10 min), and the supernatant (juice) was collected for analysis. Ten millilitres of water was added to 6 g of strawberry juice and mixed. The solution was titrated with 0.1M sodium hydroxide to obtain a pH = 8.1. Acidity was expressed in % using the formula: [(volume of NaOH × 0.1 × 0.064/6 g of juice) × 100].

#### 2.4.6. Soluble Solid Content

The soluble solid content was determined using refractometer (PAL-BX/RI, ATAGO, Tokyo, Japan) and expressed in percentage terms. The soluble solid content was determined in strawberry juice. An average of 100 g of fruit was taken from each crop in triplicate; then, the juice was removed and immediately dripped onto a refractometer slide, and the measurement was made.

#### 2.4.7. Total Anthocyanin Content

The content of anthocyanin was determined for pelargonidin-3-glucoside (PGN), which is the main anthocyanin in the fruit [29] using the method of Spayd and Morris (1981) [30,31]. A sample of 2 g of strawberry pulp was taken in three replicates from each cultivation. Eighteen millilitres of a 0.5% (*v*/*v*) solution of HCl in methanol was added to the samples. The mixture was then cooled at 4 °C for 1 h to extract PGN. After incubation, the supernatant was centrifuged and the PGN content in the sample was determined by measuring absorbance at 520 nm (maximum absorbance for anthocyanins). The PGN content was calculated from the formula (A_520_ × dilution factor × (molecular weight (MW) of PGN/molar extinction coefficient); MW_PGN_ = 433.2 and molar extinction coefficient = 2.908 × 10^4^) and expressed in mg 100 g ^−1^ FW.

#### 2.4.8. Total Soluble Phenolic Content

The total soluble phenolic content was determined using the Folin–Ciocalteu method [30,32,33]. From each cultivation, 0.5 mL of strawberry juice was taken in triplicate, and 9.5 mL of deionized water and 5 mL of diluted (1:9) Folin–Ciocalteu reagent were added to the juice. After 0.5–8 min, 4 mL of sodium carbonate solution (0.075 g mL^−1^) was added. The samples were incubated for 30 min at 30 °C and for 30 min at 4 °C; the absorbance was then measured at 760 nm. The total soluble phenolic content in the samples was determined against gallic acid standards and expressed in mg 100 g^−1^ FW.

#### 2.4.9. The Content of Vitamin C

The content of vitamin C was determined using the titration method developed by the Polish Committee for Standardization [34]. Fifty grams of fruit was weighed in triplicate from each cultivation. The samples were blended in the presence of an extraction solution and filtered. Ten millilitres of the filtrate was transferred into an Erlenmeyer flask and immediately titrated with a solution of 2,6-dichlorophenol until a pale pink colouration was obtained. The content of vitamin C was expressed in mg 100 g^−1^ FW.

#### 2.4.10. Galacturonic Acid Content

The content of galacturonic acid was determined using a Continuous Flow Analyser (CFA), SanPlus (Skalar, The Netherlands). The determination of galacturonic acid is based on the decomposition of the sample in an acidic medium. The product was transformed into furfural derivatives that react with 3-phenylphenol to form a dye that is measured at 530 nm. After hydrolysis with ammonium oxalate, the samples were prepared for determination according to the usual apparatus procedure. Three samples were taken from each crop, and the results were averaged after the determination was made.

### 2.5. FT-IR Spectra of Pectin

Pectin infrared spectra were recorded using a Nicolet 6700 FT-IR (Thermo Scientific, Waltham, MA, USA) spectrometer equipped with an ATR attachment. Lyophilized samples of pectin (three repetitions from each cultivation) derived from extraction with ammonium oxalate were used for the FT-IR studies. The spectra were recorded in the 4000–650 cm^−1^ range. Two hundred scans were performed for each measurement and then averaged with a spectral resolution of 4 cm^−1^. The spectra were corrected for baseline, normalized and analysed using OriginPro 8.5.0 SR0 software (OriginLab Corporation, Northampton, MA, USA), and the calculations were performed using Omnic software (Thermo Scientific). Calibration of the spectrometer was done by automatic subtraction of the background spectrum from subsequent measurements. Each sample was performed in triplicate. Ten spectra were made for each repetition. Then, the 30 spectra of each sample were averaged. The background was collected after 3 spectra were recorded.

### 2.6. AFM Imaging

Pectin structure was observed using a Multimode 8 atomic force microscope equipped with a Nanoscope V controller (Bruker, Billerica, MA, US) in the semiautomatic ScanAsyst^TM^ tapping mode. The samples were prepared according to the procedure described by Cybulska et al. [35]. After extraction with ammonium oxalate, the pectin were dialysed and lyophilized. After that, aqueous pectin solutions were prepared at a concentration of 0.1 mg mL^−1^ (in triplicate) and mixed for 2 h in order to separate the aggregates. Exactly 30 µL of the samples were deposited onto freshly cleaved mica using a spin coater SPIN150i (SPS-EUROPE, Putten, The Netherlands) and dried at 30 °C for 24 h. Imaging of the samples was conducted using a silicon nitride cantilever with a 2 nm nominal radius of the pyramidal tip, with a nominal resonance frequency of 130 kHz and a nominal spring constant of 0.4 N m^−1^ (ScanAsyst-AIR-HR, Bruker, Billerica, MA, USA). The following scan settings were applied: scan size, 2 × 2 µm; scan rate, 0.5 Hz (tip velocity 2 µms^−1^); and resolution 512 × 512 (3.9 nm pixel^−1^). Imaging was performed at room temperature (22 °C, relative humidity of 26–30%). Each sample was scanned a minimum of ten times. The images were processed using SPIP 6.0.14 software (Image Metrology, Hørsholm, Denmark) including flattening using third-order polynomial fitting to remove the bow effect of the surface, smoothing and noise reduction by Gaussian (SD 1) and median (3 × 3 weak) filtering. AFM images were analysed using the ‘Particle & Pore Analysis’ module of SPIP software according to the method described by Cybulska et al. (2015) [36]. Height threshold was set at 30 pm, and the segmentation of particles was performed when the minima between them were below 10 pm. Skeletonized images were then used for the determination of width, height, fibre and skeleton length of the pectin molecules. The percentage of branched fibres was calculated as the ratio of fibre length and skeleton length. All particles with fibre length below 50 nm were excluded from the analysis.

### 2.7. Rheological Properties

Rheological measurements of 1% aqueous solutions of pectin (three repetitions from each cultivation) extracted with ammonium oxalate were carried out using a Discovery HR-1 hybrid rheometer (TA Instruments, New Castle, DE, USA) with a cone-plate sensor. The measurements were carried out at a temperature of 20 ± 0.5 °C. The viscosity was measured at a constant shear rate of 10 s^−1^ (5 repetitions), while the flow curves were determined based on measurements at variable shear rates of 10 and 600 s^−1^. The Ostwald-de Waele model [37,38] was used to describe the flow curves obtained. The model is described by the following equation:

σ = kγ^n^ (1)

where σ—shear stress (Pa), k—consistency index (Pas^n^), γ—shear rate (s^−1^), n—flow behaviour index.

### 2.8. Statistical Analysis

The obtained data were analysed using STATISTICA software (Statisticav.12, StatSoft Inc., Tulsa, OK, USA). Data were analysed using a two-way analysis of variance (ANOVA) followed by a post-hoc Tukey test HSD, significant differences were determined at *p* < 0.05. The samples for the tests were prepared in no less than triplicate quantities.

## 3. Results and Discussion

Strawberries belong to the group of non-climacteric fruit, i.e., do not display a burst of autocatalytic ethylene production [39]. As a result, they stop ripening when the fruit is harvested. That is why it is so important that strawberries are harvested at the moment of optimal ripeness. In this experiment, the assessment of the ripeness of the tested Honeoye cultivar strawberries was made by the crop growers and based on a visual assessment of the skin colour, texture, flavour and aroma of the fruit. After harvest, the strawberries were immediately transferred to a cold store (4–5 °C) to determine any changes in quality. On the basis of a visual assessment (unpublished results), it was found that strawberries in cold storage had an attractive appearance for the consumer on T2 d, while on T5 d, their skin was clearly dull.

### 3.1. Fruit Quality

**Figure 2:**
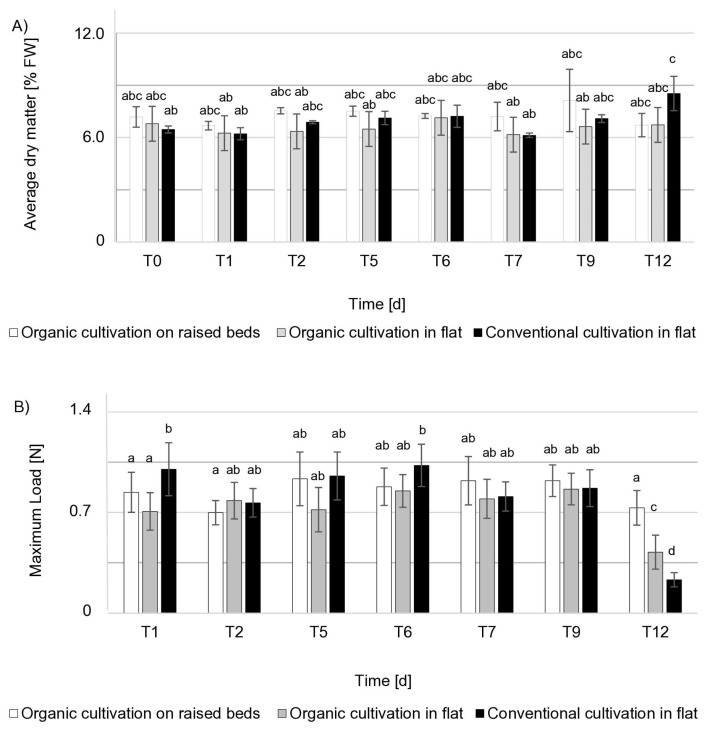
Fruit quality attributes. Average dry weight (**A**) and firmness (**B**) changes over storage time. T0—harvest day, T1–T12—days of sampling. Letters indicate the differences (a, b, c, d) between cultivation methods and storage days (*p* *<* 0.05), determined by Tukey’s HSD test. Data are means ± SD (n = 3).

Fruit size is one of the most important quality parameters for both the consumer and the fruit producer. A significant difference in fruit size was observed on the day of harvest (Table 2). The average fresh weight of the fruit collected from plants grown on raised beds (organic) was 8 g, and this was half the weight of the fruit collected from plants grown in flat-planted plots using both organic and conventional production systems. Statistically significant differences were also found between the length and width of the fruit collected from the plants grown on raised beds (organic) compared to the fruit collected from the plants grown in flat-planted plots (organic and conventional). Although the fruits from cultivated on raised beds (organic) were the smallest, they had the highest dry matter content over the course of the nine days of the experiment among the tested strawberries. On the last day of cold storage, the highest dry matter content was found in fruit harvested from plants grown in a flat-planted plot (conventional) (Figure 2). As the analysis showed, the fruit grown on raised beds (organic) was characterized by the smallest decrease in firmness (Figure 2). On the last day of cold storage, fruit firmness decreased by just 13% compared to the first day in strawberries harvested from plants grown on raised beds (organic), while for strawberries harvested from plants cultivated using the flat-planting method (organic) this decrease was on average 40%, and for fruit harvested from plants cultivated using the flat-planting method (conventional) it was 77%. This result may also be associated with the lowest average weight of fruit grown on raised beds (organic). Fruit firmness depends on the degree of adhesion between adjacent cells, cell fragility and internal turgor pressure [40]. Small fruits have a higher tissue density and are therefore firmer [41]. This function is especially important if the fruit is to be transported over a considerable distance. The harder they are, the easier it is to provide them to the contractor while maintaining good quality [42]. As predicted, the results of the maximum punching force required to penetrate the fruit varied. It should be emphasized that the cultivation of strawberry plants on raised beds (organic) had a positive impact on the firmness of the fruit.

The citric acid and soluble solid content of the fruit has a significant impact on the taste of the fruit, which is particularly important from the point of view of the consumer [43]. As predicted, there was an approximately 50% drop in overall juice acidity on T12 d as compared to T0 (Table 3). The highest acidity result was characteristic of fruit harvested from plants grown using the flat-planting method (conventional). The exception was T12 d, on which the acidity of these fruits was comparable to the acidity of fruit harvested from plants grown using the flat-planting method (organic). The decrease in citric acid content in the fruit was correlated with a slight increase in the pH of the strawberry juice. The pH of the juice remained at a similar level throughout the whole experiment, around 3.3. A slight increase in pH to 3.6–3.8 was noted on T12 d of the experiment for organic fruit (Table 3). Along with the decrease in acidity, a statistically significant decrease in the level of soluble solid content in the strawberries grown in the three ways examined was also noted (Table 3). This was influenced by two processes: on the one hand, the enzymatic degradation of cell wall polysaccharides leading to the breakdown of high-molecular-weight compounds and thus an increase in the content of simple sugars (extract) and, on the other hand, sugar metabolism by pathogens [43]. An increase in pH and a decrease in acidity are explained by metabolic changes and the consumption of organic acids in the fruit breathing process [44]. A reduction in acidity during fruit storage is a natural process resulting from the conversion of organic acids during many responses of aerobic respiration in plant cells. The decrease in the content of organic acids makes the fruit seem sweeter [45].

Anthocyanins belong to the group of phenolic compounds responsible for the colouring of fruit [46]. In addition, phenols and anthocyanins are well known for their antioxidant properties [46,47,48], and it is therefore appreciated that the content of these compounds should be kept higher. The results of the study showed that during seven days of storage, fruit from plants grown using raised beds (organic) and the flat-planting method (organic) had a significantly higher (by 54% on average) accumulation of anthocyanins than fruit from plants grown using the flat-planting method (conventional) (Table 3). The increase in anthocyanin content could be attributed to their release due to the breakdown of cellular components [48]. The method and conditions of cultivation may affect the activity of the enzymes (for example, phenylalanine ammonium lyase, chalcone synthase, chalcone isomerase) responsible for the synthesis of anthocyanins. Destruction of the enzyme or the degradation of the cell structure leads to the disruption of anthocyanin biosynthesis and reduction of fruit quality. The lower yields of anthocyanin-synthesizing enzymes were particularly evident in fruit cultivated in a flat-planted plot (conventional) [49].

The method and conditions of cultivation were not an important factor in determining the content of phenols in strawberries (Table 3). Statistically significant differences were observed between the phenol contents of the strawberries on individual days. It is claimed that the degradation of the cellular structure at the stage of fruit ageing may be responsible for fluctuations in the phenol content [50]. With phenols being more concentrated in the outer parts of cells than in vacuoles, the increase in phenolic compounds may be attributed to their release due to the breakdown of cellular components. On the other hand, the reduction of phenols can be attributed to their enzymatic degradation. Cell damage may also cause the release of oxidizing and hydrolytic enzymes [49]. The increase in phenolic compound content on T12 d compared to T9 d is consistent with the results of other studies [51] and is explained by the development of new compounds with antioxidant capacity that increase the content of phenols [52].

Vitamin C plays a significant role in human nutrition, although it is only a minor constituent of fruit and vegetables [44]. Vitamin C (ascorbic acid) is usually considered as an indicator of the quality of nutrients during food processing and storage because it is rapidly degraded due to, among other things, light, pH, temperature or crushing of fruit tissue. It may be observed that if ascorbic acid is well preserved, other nutrients are also well preserved, except for anthocyanins, the degradation of which is accelerated by ascorbic acid [53]. As expected, storage led to vitamin C degradation in the fruit, but the method of cultivation was not a differentiating factor (Table 3). On the day of harvest, the content of vitamin C was on average 58.7 mg in 100 g^−1^ FW, while on the last day of the experiment, the average was 26.8 mg in 100 g^−1^ FW for the tested strawberries from three different crops. Strawberries are considered one of the fruits most rich in ascorbic acids. Fresh fruit is assumed to contain an average of 50 mg in 100 g^−1^ FW of vitamin C [53,54]. One of the reasons for the loss of vitamin C during storage is its oxidation due to contact with air. The microorganisms responsible for the rotting and decomposing processes cause cell wall degradation. Cell walls are a natural protective layer that controls the penetration of oxygen and carbon dioxide. The consequence of degradation is the increase in vitamin C self-oxidation intensity [55,56]. In addition, ascorbic acid in strawberry fruit can be synthesized from D-galacturonic acid, the main component of cell-wall pectin [57]. The building blocks of pectin are the D-galacturonic acid subunits, to which various sugar residues are attached [58]. The release of D-galacturonic acid occurs as a result of the enzymatic hydrolysis of the pectin chain. Reducing the level of vitamin C during storage may be due to the loss of integrity of strawberry cell-wall structures and the release of D-galacturonic acid subunits [59]. In order to confirm this relationship, hydrolysis with ammonium oxalate was carried out and the content of D-galacturonic acid in the examined fruit was determined. On average, a 13.12% decrease in D-galacturonic acid content on the twelfth day of storage (Table 3) compared to the harvest day was observed for fruit harvested from plants grown on raised beds (organic), which confirms that the decrease in vitamin C content during storage is correlated with the decrease in D-galacturonic acid content. For strawberries harvested from plants grown in flat-planted plots (both organic and conventional production systems), the galacturonic acid content on T0 d was 3.53% and 1.83% lower, respectively, compared to T12 d. It should be emphasized that a decrease in the content of D-galacturonic acid is not the only reason for a decrease in the level of ascorbic acid in the tested fruit. The decrease in vitamin C levels may also be due to an increase in ascorbinase enzyme activity [45].

### 3.2. FT-IR Spectra

**Figure 3:**
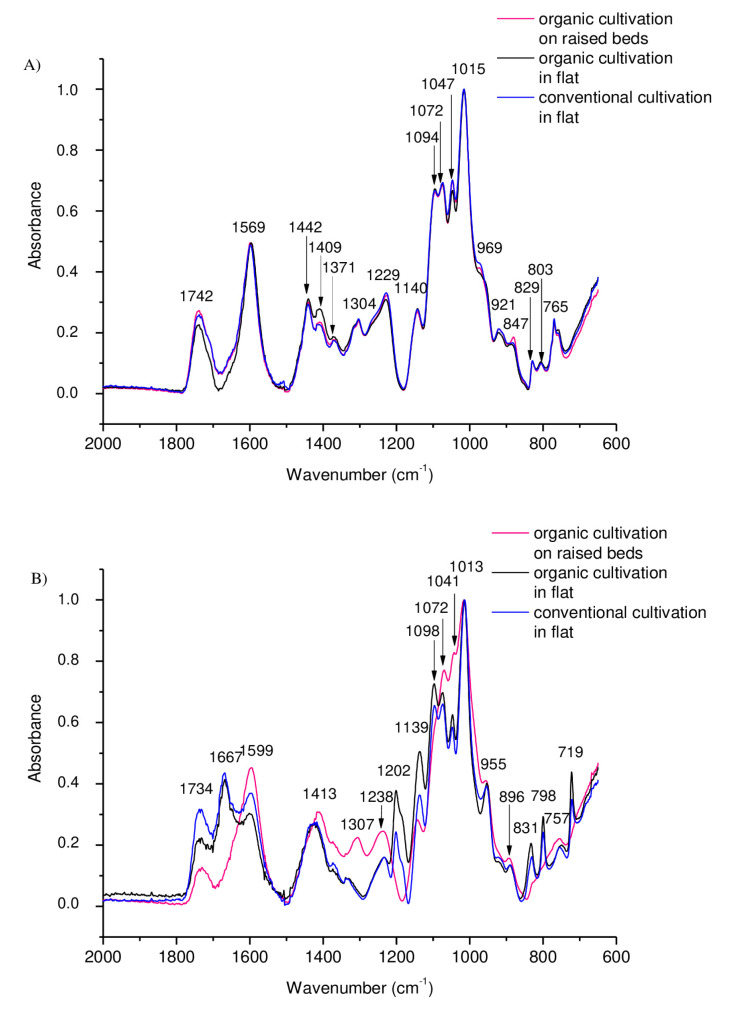
FT-IR spectrum of pectin after extraction with ammonium oxalate from strawberries cv. Honeoye cultivated in three different ways on (**A**) the day of harvest (T0) and (**B**) the twelfth day of storage (T12).

The FT-IR spectra recorded for the T0 d samples reflect the chemical structure of the pectin extracted from the strawberries (Figure 3A). The almost completely overlapping spectra for T0 d samples suggests that the cultivation method had no effect on the pectin structure. The fingerprint region for the pectin samples occurs in the range of 1000–1800 cm^−1^. The bands at 1742 cm^−1^ are characteristic of the esterified carboxyl groups in pectin. The peak at 1569 cm^−1^ indicates the occurrence of asymmetric stretching vibrations of the COO^−^ group characteristic of pectin [59]. Other characteristic bands testifying to the presence of pectin occur at 829 cm^−1^, 969 cm^−1^, 1015 cm^−1^, 1229 cm^−1^, 1409 cm^−1^ and 1442 cm^−1^ [60,61]. Nevertheless, there are also bands that can be attributed to the presence of several polysaccharides. An example is the 1371 cm^−1^ peak, which is characteristic of both cellulose and hemicellulose, and probably due to a similar molecular composition [62].

As expected, significant differences in the pectin structure were observed on T12 d of the fruit in cold storage (Figure 3B). These differences are particularly evident in the intensity of some bands. The spectrum of the pectin of strawberries from plants grown using the flat-planting method (conventional) differs significantly from the spectra that reflect the structure of the pectin of fruit derived from plants grown in an organic way. In the wall structure of strawberries harvested from plants grown using the flat-planting method (conventional), a decrease in the peak of 1734 cm^−1^ was observed compared to the spectra of the pectin structure of fruit from organic farming. Peaks from strawberries harvested from plants grown using the flat-planting method (conventional) (1599, 1413, 1307, 1238 cm^−1^) showed a greater intensity compared to the spectra of the pectin structure of fruit from organic farming. In the pectin structure of strawberries from plants grown in a flat-planted plot (conventional), no peaks were observed at 1667 and 1202 cm^−1^ [63]. The absence of these peaks may be attributed to the enzymatic degradation of galacturonic acid chains and the decarboxylation of pectin, which are one of the consequences of fungal and bacterial infections.

### 3.3. AFM Imaging

**Figure 4:**
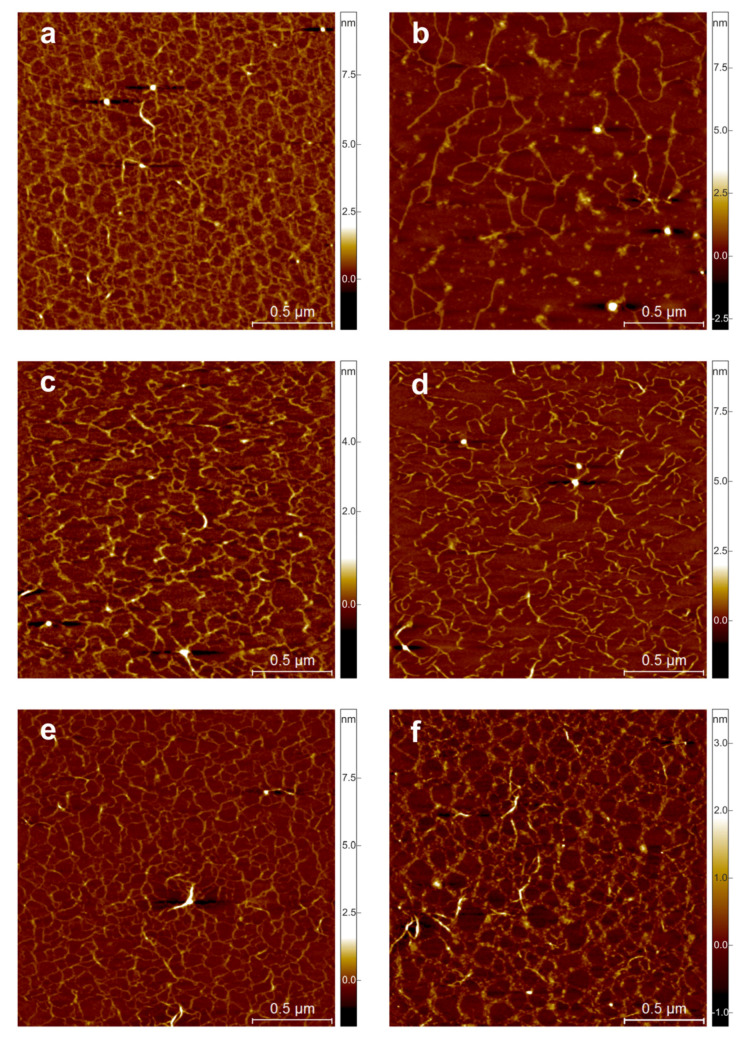
AFM height images of pectin extracted with ammonium oxalate during cold storage; (**a**)—organic cultivation on raised beds T0, (**b**)—organic cultivation on raised beds T12, (**c**)—organic cultivation in a flat-planted plot T0, (**d**)—organic cultivation in a flat-planted plot T12, (**e**)—conventional cultivation in a flat-planted plot T0, (**f**)—conventional cultivation in a flat-planted plot T12.

The nanostructure of pectin extracted with ammonium oxalate observed with AFM is shown in Figure 4. The pectin derived from the fruit on the harvest day (T0 d) had a regular, crosslinked structure. It was noted that the height of pectin fibrils increased with storage time, especially in the case of cultivation on raised beds (organic), where height was almost doubled after storage time (Table 4). Based on a visual examination of the AFM images, it appears that pectin from strawberries grown in a flat-planted plot (organic) had a less organized structure compared to the other samples tested on T0 d (Figure 4a,c,e). This pectin was also characterised by a significantly lower content of long molecules >5000 nm (Table 4). This may explain why the viscosity of pectin from fruit grown in a flat-planted plot (organic) reaches the lowest values compared to the other methods used (Table 5). During cold storage of strawberries, the pectin network structure underwent disorganization and destruction, which was especially noticeable in samples derived from fruit grown in a flat-planted plot (organic) and on raised beds (organic) (Figure 4d,f). The content of side branches in total skeleton length of pectin molecules has decreased in all samples during storage. Pectin isolated from strawberries grown in a flat-planted plot (organic) contained a considerable amount of non-branched polymers and demonstrated the highest scale of debranching processes in comparison to the other samples, which is shown by the lowest content of side branches in total skeleton length in T0 and T12 d. On T12 d, fibrils with a length of 1000–5000 nm for strawberries grown in a flat-planted plot (organic) and above 5000 nm for fruit grown in a flat-planted plot (conventional) and on raised beds (organic) dominated (Table 4). The pectin derived from strawberries grown in a flat-planted plot (conventional) showed a relatively organized structure on T12 d, which may be attributed to the activity of artificial fertilizers, which could inhibit pectin-degrading enzymes. Significant differences in the pectin structure on T12 d were also confirmed by FT-IR spectra (Figure 3). Studies show that the destruction of the regular structure of pectin derived from fruit during storage is a natural process [63]. Disassembly of the pectin network extracted from the stored strawberries of the organic cultivar was reflected in the very low viscosity of T12 d. Maintenance of the crosslinked pectin in T12 d in the case of pectin extracted from conventionally grown strawberries also influenced the rheological properties of the polysaccharide. The viscosity of these pectins was the highest among the samples measured and decreased only slightly during cold storage. Thus, conventional farming may still be regarded as the most appropriate technique for the technological usage of stored strawberries.

### 3.4. Rheological Properties of Pectin Extracted from Strawberries

Pectin derived from strawberries are commonly used in industry, and their rheological properties determine their application. Pectin can be used to thicken paints and pastes as well as in the manufacture of medicines [64]. Due to their natural origin, pectins are used in the food industry as a thickener, stabilizer and flavour enhancer [65]. It should be emphasized that when pectins are used as a gelling and thickening agent, they should improve the texture of food products. Higher viscosity indicates the greater ability of pectin to thicken the solution [38].

The viscosity of 1% aqueous pectin solutions was measured. The pectins were extracted from fruit with ammonium oxalate (procedure in Materials and Methods). This method made it possible to obtain all of the pectin domains found in strawberry. Statistically significant differences were observed in the viscosity of 1% pectin solutions depending on the date of the harvest and the way in which the strawberries were grown. The solutions on T0 d showed a much higher viscosity than the solutions on T12 d. The highest viscosity on T0 d and T12 d was characteristic of a pectin solution derived from strawberries harvested from plants growing in flat-planted soil (conventional) (Table 5). It should be noted that the viscosity of a 1% pectin solution from strawberries harvested from plants grown on raised beds (organic) on T0 d was comparable to the viscosity of a 1% pectin solution derived from strawberries harvested from plants grown in a flat-planted plot (conventional) on T12 d (Table 5). The decrease in viscosity may be attributed to the enzymatic activity of microorganisms that degrade the fruit cell wall material [13]. The changes in viscosity are influenced by the pectin molecular weight, pectin methylation degree, solution pH, counter-ion concentration and temperature [15]. The results of the viscosity measurement show an insufficient protection of strawberry plants by organic preparations.

**Figure 5:**
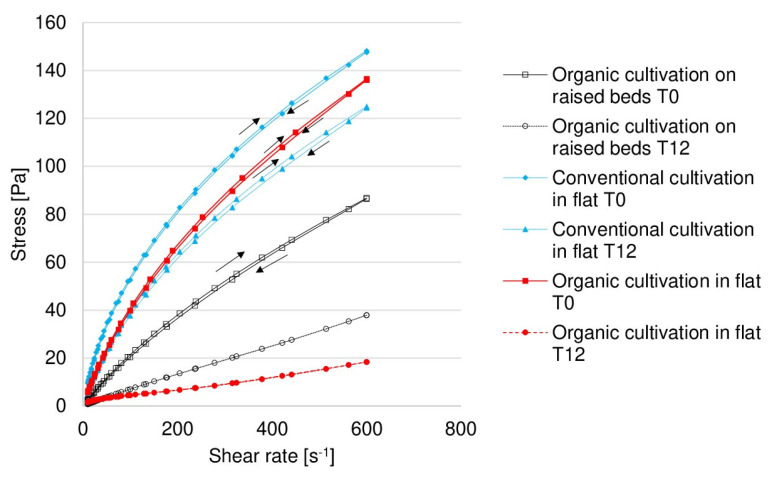
Flow curves for pectin samples extracted with ammonium oxalate. The arrow indicates the direction of the line. T0—harvest day, T12—last day of the experiment.

Downward and upward flow curves were collected for the samples tested and fitted to the Ostwald–de Waele model (Figure 5). The model allows determining the rheological properties of the tested solutions. Curves for 1% pectin solutions derived from strawberries harvested from plants grown on raised beds (organic) T0, in flat-planted soil(organic) T0 and T12, in flat-planted soil (conventional) T0 and T12 are very well described by the model because R^2^ > 0.99. R^2^ for strawberry pectin solutions derived from fruit harvested from plants grown on raised beds (organic) T12 was slightly lower but still higher than 0.97. The consistency index k contained in Ostwald–de Waele model is related to the viscosity of the samples. A higher k index means a higher viscosity. The k index confirmed that 1% solutions of pectin derived from strawberries harvested from plants cultivated in an organic way were characterized by a much lower viscosity on the T0 d and T12 d of the experiment compared to solutions of pectin derived from strawberries cultivated using the flat-planting method (conventional) (Table 5). However, in comparison with the viscosity of pure water (0.001 Pas), the tested solutions showed at least a ten times higher viscosity than water.

Another important parameter of the Ostwald-de Waele model is the melt flow index n, which allows liquids to be characterized as Newtonian (n = 1) or non-Newtonian (n < 1, n > 1) [66].

Pectin solutions derived from strawberries harvested from organic and conventional cultivated plants were characterized as shear-thinning liquids (n < 1, pseudoplastic). Shear-thinned fluids are liquids whose viscosity decreases as the shear rate increases; pseudoplastic is the term which describes such fluids. The lower the n-factor, the greater the fluid’s pseudoplasticity [66]. Solutions of pectin from crops grown on raised beds (organic) T12 and in a flat-planted plot (conventional) T0 were characterized by the highest levels of pseudoplasticity. These pseudoplastic fluids may be used in the production of beverages at the stage of filling, mixing, pumping [66,67] and in pharmacology to create drug carriers or syrups, and they are also used to produce anti-inflammatory and gastroprotective medicines [64].

In four of the tested samples—those grown in flat-planted plots (organic T0, conventional T0, conventional T12) and on raised beds (organic) T0—the downward and upward curves did not coincide and small hysteresis loops were obtained (Figure 5), which indicates their rheopectic behaviour [68,69]. In this case, the hysteresis loop area does not change with the type of sample. The flow curves for these samples show non-Newtonian plastic-type behaviour. A certain amount of shear stress must be applied to the fluid before creating a flow effect [69]. No hysteresis loop was observed for the pectin solutions derived from organic strawberries (flat-planting method (organic) T12, raised beds (organic) T12). It was probably the case that, on T12 d, microorganisms led to the complete degradation of the pectin structure in the case of samples derived from organic fruit (flat-planting (organic) T12, raised beds (organic) T12). The lack of a hysteresis loop confirms that the protective effect of organic preparations is not as long-lasting as conventional preparations [70].

## 4. Conclusions

Strawberries produced by organic farming methods had better nutritional composition compared to conventional fruit. The differences were particularly notable with regard to the anthocyanin content, which is well known for its antioxidant properties, but increased anthocyanin content also results in preferable strawberry colour. The organic fruit also had better sensory properties because of its lower acidity and higher soluble solid content. These properties are particularly relevant to consumers. The loss of fruit firmness and rheological properties was closely related to the degradation of the pectin structure. Organic strawberry pectin was more rapidly degraded than conventional strawberry pectin. The rapid hydrolysis of the pectin structure limits the possibilities for their processing and use as gelling or stabilizing agents. It seems that the implementation of ecological cultivation methods ensures that strawberries with superior taste and health values are obtained compared to conventional methods. On the other hand, conventional methods allow for longer maintenance of the cross-linked pectin structure, which translates into longer preservation of relatively stable viscosity of pectin solutions.

## Figures and Tables

**Table 1:** Preparations used in the cultivation of strawberries cv. Honeoye.

Organic Cultivation on Raised Beds	Organic-Cultivation–Flat-Planting Method	Conventional-Cultivation–Flat-Planting Method
Leżachów Osada (N 50.12972, E 022.62469)	Rozbórz (N 50.0548563, E 022.5451595)	Jawornik Polski (N 49.95751 E 022.54519)
**Soil preparations:** Polyversum WP (Bio Agris, Poland) (150 g ha^−1^) Amylo-X (Certis, UK) (2.5 kg ha^−1^) Vaxiplant (Galvet, Poland) (1 L ha^−1^) Serenade ASO (Bayer, UK) (8 L ha^−1^) Beta-Chikol (Poli-Farm Company, Poland) (35 L ha^−1^) Wetcit (BICONT, Poland) (10 L ha^−1^) VitiSan (Biofa AG, Germany) (16 L ha^−1^; main component is KHCO_3_ in dose 19 g ha^−1^) Lecitec (Target S.A., Poland) (16 L ha^−1^) Megis (ICBpharma, Poland) (3.5 L ha^−1^; main component is Si in dose 0.04 g ha^−1^) EmFarma Plus (ProBiotics, Poland) 920 L ha^−1^) Humus 1 (Agrecol, Poland) (20 L ha^−1^) biFosfor (Agrarius, Poland) (1 kg ha^−1^) Biprotect (AEF Global INC, Canada) (1 kg ha^−1^) **Pest control:** Naturalis L (Fargro, UK) (2 L ha^−1^) Wetcit (Biocont, Poland) (3.5 L ha^−1^) Wetcit(Biocont, Poland) (14 L ha^−1^) Microcat Flic (Vitera, Suwałki, Poland) (14 L ha^−1^; main component is K_2_O in dose 0.8 g ha^−1^) Microcat Bon (Vitera, Suwałki, Poland) (0.15 L ha^−1^; main component is P_2_O_5_ in dose 0.12 g ha^−1^)	**Soil preparations:** Condit (JSC ‘JuknevičiausKompostas’, Vilnius, Lithuania) (2 t ha^−1^; main component is N in dose 20 kg ha^−1^) Potassium sulfate (AMPOL-MEROL, Poland) (300 kg ha^−1^) Physio Natur PKS (TimacAgro, Romania) (1 t ha^−1^ main components are CaO in dose 0.28 t ha^−1^, P_2_O_5_ in dose 0.13 t ha^−1^, K_2_O in dose 0.15 t ha^−1^, MgO in dose 0.02 t ha^−1^ and SO_3_ in dose 0.19 t ha^−1^) **Foliar preparations:** Mc Calcium (BiodevasLaboratoires, France) (1 L ha^−1^; main component is Ca in dose 0.07 g ha^−1^) Calcio Sprint (Martinez and Valdivieso, Chile) (6 L ha^−1^; main component is water-soluble CaO 0.5 g ha^−1^) Megafol (Valagro, USA) (2 L ha^−1^; main components are N ain dose 0.05 g ha^−1^ and K_2_O in dose 0.2 g ha^−1^) Olibio (Biodevas, Spain) (3 L ha^−1^; main components are Cu, Fe, Mn, Zn in dose 0.02 g ha^−1^) Activstart (BiodevasLaboratoires, France) (2 L ha^−1^) Ecovigor (TimacAgro, Spain) (5 L ha^−1^; main components are N in dose 0.2 g ha^−1^ and K_2_O in dose 0.3 g ha^−1^)	**Soil preparations:** PERLKA (Alz Chem, Germany) (300 kg ha^−1^; main components are N in dose 59 kg ha^−1^ and CaO in dose 150 kg ha^−1^) Captan (Bonide, New York, USA) (2.1 kg ha^−1^) Switch 62,5 WG (Syngenta, Basel, Switzerland) (0.75 kg ha^−1^) Signum 33 WG (Syngenta, Basel, Switzerland) (1 kg ha^−1^) Luna Sensation (Bayer, UK) (0.7 L ha^−1^) Amylo-X (Fitogest, Italy) (2.5 kg ha^−1^) Serenade Max (Bayer, UK) (8 L ha^−1^) **Foliar preparations:** Mc Calcium (BiodevasLaboratoires, France) (1 L ha^−1^; main component is Ca in dose 0.07 g ha^−1^) Megafol (Valagro, USA) (2 l ha^−1^; main components are N ain dose 0.05 g ha^−1^ and K_2_O in dose 0.17 g ha^−1^) Potassium phosphide (AMPOL-MEROL, Poland) (1.5 kg ha^−1^) Olibio (Biodevas, Spain) (2 L ha^−1^; main component are Cu, Fe, Mn, Zn in dose 0.01 g ha^−1^) Humus 1 (Agrecol, Poland) (10.5 L ha^−1^) **Pest control:** Mospilan 20 SP (SumiAgro, Durham, USA) (0.125 kg ha^−1^) SpinTor 240 EC (Corteva, Warsaw, Poland) (0.15 L ha) Microcat Ole (Vitera, Suwałki, Poland) (1.5 L ha^−1^; main component is K_2_O in dose 0.1 g ha^−1^) Microcat Flic (Vitera, Suwałki, Poland) (2 L ha^−1^; main component is K_2_O in dose 0.1 g ha^−1^)

**Table 2:** Fruit dimensions on the day of harvest.

	Organic Cultivation on Raised Beds	Organic-Cultivation–Flat-Planting Method	Conventional-Cultivation–Flat-Planting Method
Fresh weight [g]	8.1 ^a^ ± 0.4	16.6 ^b^ ± 0.7	16.6 ^b^ ± 2.3
Length [mm]	26.5 ^a^ ± 2.7	34.5 ^b^ ± 3.2	36.9 ^c^ ± 3.1
Width [mm]	26.8 ^a^ ± 4.0	34.5 ^b^ ± 4.5	36.3 ^b^ ± 3.8

Different letters (a, b, c) indicate significant differences between the cultivation methods (*p* < 0.05), determined by Tukey’s HSD test. Data are means ± SD (n = 50).

**Table 3:** Quality results of the strawberries tested. T0—harvest day, T1–T12—days of sampling.

		T0	T1	T2	T5	T6	T7	T9	T12
Average acidity [%]	Organic cultivation on raised beds	1.2 ^a^ ± 0.0	1.1 ^a^ ± 0.0	1.0 ^bd^ ± 0.0	0.8 ^bcde^ ± 0.0	0.8 ^bde^ ± 0.0	0.8 ^bd^ ± 0.0	0.9 ^bd^ ± 0.0	0.6 ^ce^ ± 0.0
	Organic-cultivation–flat-planting	1.1 ^ab^ ± 0.0	0.9^abd^ ± 0.0	0.9 ^abd^± 0.0	0.6 ^cde^ ± 0.0	0.7 ^cde^ ±0.0	0.7 ^bd^ ± 0.0	0.7 ^bd^ ± 0.0	0.5 ^ce^ ± 0.0
	Conventional-cultivation–flat-planting	1.3 ^a^ ± 0.0	1.0 ^a^ ± 0.1	1.1 ^bd^ ± 0.0	0.7 ^bd^ ± 0.0	0.8 ^bd^ ± 0.0	0.8 ^bd^ ± 0.0	0.9 ^bd^ ± 0.0	0.7 ^e^ ± 0.0
pH [-log H+]	Organic cultivation on raised beds	3.4 ^a^ ± 0.0	3.2 ^ab^ ± 0.1	3.3 ^abc^± 0.1	3.2 ^ab^ ± 0.0	3.4 ^ac^± 0.0	3.2 ^ab^± 0.0	3.3 ^ab^± 0.0	3.6 ^ad^± 0.0
	Organic-cultivation– flat-planting	3.4 ^ab^ ± 0.0	3.2 ^ac^ ± 0.0	3.4 ^a^± 0.0	3.4 ^ad^ ± 0.1	3.4 ^ab^ ± 0.1	3.4 ^a^ ± 0.0	3.3 ^acd^ ± 0.0	3.8 ^ae^ ± 0.0
	Conventional-cultivation–flat-planting	3.3 ^ab^ ± 0.0	3.2 ^ad^ ± 0.0	3.2 ^ad^ ± 0.1	3.2 ^ad^ ± 0.0	3.4 ^abc^ ± 0.0	3.3 ^acd^ ± 0.0	3.3 ^ac^ ± 0.0	3.2 ^ad^ ± 0.1
Average soluble solid content [%]	Organic cultivation on raised beds	6.8 ^a^ ± 0.0	6.3 ^ab^± 0.0	6.6 ^a^ ± 0.0	6.7 ^a^ ± 0.0	6.3 ^a^ ± 0.0	6.7 ^a^ ± 0.0	6.4 ^a^ ± 0.0	5.3 ^c^ ± 0.0
	Organic-cultivation–flat-planting	6.4 ^a^ ± 0.0	5.9 ^bc^ ± 0.0	5.5 ^c^ ± 0.0	5.3 ^c^ ± 0.0	5.7 ^c^ ± 0.0	6.4 ^a^ ± 0.0	6.3 ^a^ ± 0.0	4.7 ^d^ ± 0.1
	Conventional-cultivation–flat-planting	6.3 ^a^ ± 0.0	6.1 ^bc^ ± 0.0	6.2 ^a^ ± 0.0	5.7 ^bc^ ± 0.1	5.8 ^cb^ ± 0.0	5.7 ^c^ ± 0.0	6.7 ^a^ ± 0.0	5.7 ^c^ ± 0.0
Average anthocyanin content [mg 100 g^−1^ FW]	Organic cultivation on raised beds	33.1 ^ab^± 2.8	29.4 ^a^ ± 0.4	29.5 ^a^ ± 1.4	29.6 ^a^ ± 1.1	36.3 ^a^ ± 1.9	40.2 ^a^ ± 5.3	15.7 ^c^ ± 0.8	11.2 ^c^ ± 2.6
	Organic-cultivation–flat-planting	34.0 ^ad^± 4.0	30.0 ^a^ ± 1.7	32.5 ^a^ ± 4.0	37.2 ^a^ ± 1.5	32.9 ^bd^ ± 1.0	31.5 ^a^ ± 2.1	29.2 ^d^ ± 9.0	15.4 ^cb^ ± 0.8
	Conventional-cultivation–flat-planting	12.2 ^c^ ± 0.9	14.1 ^c^ ± 0.6	14.7 ^c^ ± 0.7	11.7 ^c^ ± 1.6	19.9 ^c^ ± 3.3	15.2 ^c^ ± 0.8	14.8 ^c^ ± 4.0	12.9 ^cabc^ ± 0.6
Average soluble phenolic content [mg 100 g^−1^ FW]	Organic cultivation on raised beds	124.3 ^a^ ± 0.1	142.8 ^b^ ± 0.1	127.2 ^a^ ± 1.0	136.9 ^b^ ± 0.2	143.4 ^b^ ± 0.3	144.9 ^b^ ± 0.4	111.2 ^c^ ± 0.1	142.3 ^b^± 0.2
	Organic-cultivation–flat-planting	109.2 ^c^ ± 0.2	125.4 ^a^ ± 0.2	114.4 ^ac^ ± 0.6	132.7 ^ab^ ± 0.2	127.5 ^a^ ± 0.3	131.4 ^a^ ± 0.5	89.4 ^d^ ± 0.3	130.3 ^a^ ± 0.8
	Conventional-cultivation–flat-planting	103.8 ^cd^ ± 0.2	119.1 ^ac^ ± 0.2	106.7 ^cd^ ± 0.4	129.2 ^a^ ± 0.6	95.9 ^d^ ± 0.1	123.1 ^a^ ± 0.2	104.7 ^cd^ ± 0.3	126.5 ^a^ ± 0.3
Average vitamin C content [mg 100 g^−1^ FW]	Organic cultivation on raised beds	63.4 ^a^ ± 2.2	nd	62.3 ^ab^ ± 0.4	nd	37.4 ^c^ ± 0.5	nd	30.2 ^d^ ± 1.8	nd
	Organic-cultivation in–flat-planting	59.9 ^ae^± 0.6	nd	58.8 ^be^ ± 0.6	nd	38.6 ^c^ ± 0.6	nd	24.8 ^f^ ± 1.8	nd
	Conventional-cultivation–flat-planting	52.9 ^g^ ± 0.6	nd	65.3 ^a^ ± 1.2	nd	36.7 ^c^ ± 0.8	nd	25.5 ^f^ ± 2,8	nd
Galacturonic acid content [%]	Organic cultivation on raised beds	53.6 ^a^ ± 1.3	nd	nd	nd	nd	nd	nd	40.4 ^c^ ± 1.3
	Organic-cultivation–flat-planting	55.8 ^b^ ± 3.6	nd	nd	nd	nd	nd	nd	59.3 ^d^ ± 2.5
	Conventional-cultivation–flat-planting	53.3 ^a^ ± 3.2	nd	nd	nd	nd	nd	nd	55.2 ^ab^ ± 1.3

Letters indicate (a, b, c, d, e, f, g) the differences between cultivation methods and storage days (*p* < 0.05), determined by Tukey’s HSD test. Data are means ± SD (n = 3); nd—not determined.

**Table 4:** Topographic parameters of pectin molecules extracted from fresh (T0) and stored (T12) strawberries with ammonium oxalate.

Sample	Average Height [nm]	% of Branches in Total Skeleton Length	The Percentage of Pectin Molecules Characterized by Different Fibre Lengths (% of Total Fibre Length)				
			Range of Fibre Length [nm]				
			0–100	100–500	500–1000	1000–5000	>5000
Organic cultivation on raised beds T0	0.42 ± 0.28 ^c^	89.1	5.5	17.1	11.6	1.8	64.1
Organic cultivation on raised beds T12	0.74 ± 0.37 ^d^	85.8	5.8	6.6	9.1	36.1	42.4
Organic-cultivation–flat-planting T0	0.30 ± 0.16 ^ab^	66.4	10.1	48.0	21.3	6.7	13.9
Organic-cultivation–flat-planting T12	0.47 ± 0.21 ^c^	55.8	6.5	32.0	14.1	39.1	8.3
Conventional-cultivation–flat-planting T0	0.26 ± 0.02 ^a^	85.2	6.2	22.8	8.2	15.8	47.0
Conventional-cultivation–flat-planting T12	0.33 ± 0.24 ^b^	80.8	5.8	21.6	13.7	22.5	36.4

Letters indicates the differences (a, b, c, d) between cultivation methods and storage days (*p* < 0.05), determined by Tukey’s HSD test. Data are means ± SD (n = 3).

**Table 5:** Parameters of the Ostwald–de Waele model describing the rheological properties of 1% pectin solutions.

Sample	Viscosity [Pas]	Upward Curve			Downward Curve		
		k	n	R^2^	k	n	R^2^
Organic cultivation on raised beds T0	0.40 ^a^ ± 0.01	0.75 ^a^ ± 0.04	0.81 ^a^ ± 0.01	0.997	0.88 ^a^ ± 0.09	0.79 ^a^ ± 0.01	0.997
Organic cultivation on raised beds T12	0.03 ^b^ ± 0.01	0.29 ^b^ ± 0.09	0.57 ^b^ ± 0.02	0.979	0.35 ^ab^ ± 0.10	0.53 ±0.02	0.982
Organic-cultivation–flat-planting T0	0.21 ^c^ ± 0.00	0.10 ^b^ ± 0.01	0.97 ^c^ ± 0.01	1.000	0.12 ^ab^ ± 0.01	0.95 ^c^ ± 0.01	1.000
Organic-cultivation–flat-planting T12	0.07 ^b^ ± 0.01	0.05 ^b^ ± 0.00	0.99 ^c^ ± 0.01	1.000	0.05 ^b^ ± 0.01	0.98 ^c^ ± 0.01	1.000
Conventional-cultivation–flat-planting T0	0.51 ^ad^ ± 0.03	2.67 ^c^ ± 0.26	0.68 ^d^ ± 0.01	0.995	2.92 ^c^ ± 0.38	0.67 ^d^ ± 0.01	0.995
Conventional-cultivation–flat-planting T12	0.40 ^a^ ± 0.03	1.75 ^d^ ± 0.23	0.74 ^e^ ± 0.01	0.996	2.36 ^c^ ± 0.57	0.70 ^d^ ± 0.03	0.997

k—consistency index (Pas^n^); n—flow behaviour index; R^2^—determination coefficient. Letters (a, b, c, d) indicate the differences between cultivation methods and storage days (between T0 and T12) (*p* < 0.05), determined by Tukey’s HSD test. SD indicates the standard deviation of selected parameters of the Ostwald-de Waele model from three replicates.
